# Spontaneous brain-wide rotational dynamics constrain cue-evoked behavior

**DOI:** 10.64898/2026.10.17.759884

**Published:** 2026-10-21

**Authors:** Sihan Chen, Yunlong Xu, Doruk Efe Gökmen, Michel Fruchart, Brent Doiron, Vincenzo Vitelli

## Abstract

Uncovering low-dimensional representations of brain-wide neural dynamics and their relationship to behavior remains a central challenge. Here, we show that spontaneous brain-wide rotational dynamics constrain cue-evoked behavior and learning. To systematically establish these findings, we developed a dimensionality-reduction technique called Rotational Component Analysis (RCA) that optimizes the detection of low-variance rotational modes even within individual trials. We applied RCA to spike train data from motor cortices of monkeys and humans, mouse cortex-wide calcium imaging and in human functional magnetic resonance imaging (fMRI) and magnetoencephalography (MEG). In mice, a rotational mode identified from activity recorded at rest predicts cue-evoked behavior: the phase of rotation when a randomly timed cue arrives predicts whether the animal subsequently pulls the lever. We explain this surprisingly strong correlation using both a recurrent neural network (RNN) and a minimal two-dimensional model of response learning, in which the intrinsic rotational mode serves as a dynamical constraint on response and learning. We further identify double-rotation modes containing two centers of rotation that separate preparatory from task dynamics in both mouse and human data. Remarkably, corresponding modes also organize neural dynamics during a non-motor human task, and rotational irreversibility captures early psychosis even when a principal component analysis shows no group difference. Our work establishes rotational dynamics as low-dimensional coordinates that constrain how ongoing brain states are converted into behavior. More broadly, RCA provides a versatile tool for detecting hidden nonreciprocal dynamics in biological and physical datasets.

